# Distal Traditional Acupuncture Points of the Large Intestinal Meridian and the Stomach Meridian Differently Affect Heart Rate Variability and Oxygenation of the Trapezius Muscle

**DOI:** 10.1155/2014/283010

**Published:** 2014-02-19

**Authors:** Yukiko Shiro, Young-Chang P. Arai, Tatsunori Ikemoto, Takashi Kawai, Masahiko Ikeuchi, Takahiro Ushida

**Affiliations:** ^1^Department of Physical Therapy, Faculty of Rehabilitation Science, Nagoya Gakuin University, 1350 Kamishinanocho, Seto, Aichi 480-1298, Japan; ^2^Multidisciplinary Pain Centre, School of Medicine, Aichi Medical University, 21 Karimata, Nagakutecho, Aichigun, Aichi 480-1195, Japan

## Abstract

Physicians in traditional Chinese medicine have found that acupoints and meridians have effects on specific parts of the body. The aim of this study was to see how acupressure at distal acupuncture points of a specific meridian affects heart rate variability (HRV) and oxygenation of the trapezius muscle. Forty-one female participants were randomly allocated to three groups. Subjects in the Stomach Meridian acupuncture point (ST) group received acupressure at ST 34, ST 36, and ST 41, subjects in the Large Intestinal Meridian acupuncture point (LI) group received acupressure at LI 4, LI 10, and LI 11, and subjects in the control group did not receive any stimuli. HRV and oxygenation of the trapezius muscles were measured. The high frequency components of HRV in the control and LI groups tended to be higher than those in the ST group. Total hemoglobin in the control and LI groups eventually reached significantly higher levels than in the ST group. While oxyhemoglobin (ΔO_2_Hb) in the control and LI groups did not change, ΔO_2_Hb in the ST significantly decreased temporarily.

## 1. Introduction

Distal traditional acupuncture points, “Hegu” (LI 4), “Shousanli” (LI 10), and “Quchi” (LI 11), are contained in the Large Intestinal Meridian and have been suggested to be the particular points for improving neck-shoulder-arm disorder. Chronic neck and shoulder pain is a very common symptom especially in females. Subjects with neck and shoulder pain showed impaired regulation of microcirculation in the trapezius muscle [1, 2] and impaired function of sympathetic nerves [3]. Sympathetic nerve activity contributes to vasoconstriction and exerts control over the skeletal muscle vasculature [4, 5]. Mechanical pressure such as massage and acupressure has been known to reduce musculoskeletal pain, promote relaxation, and increase regional blood circulation and parasympathetic nervous activity [6, 7]. Our previous study showed that acupressure at these distal acupuncture points improved pain conditions and influenced autonomic nervous activity in females with chronic neck pain [8].

We hypothesized that distal acupuncture points in each meridian would have specific effects, thereby differently influencing autonomic nervous activity and muscle blood flow and oxygenation. The aim of the present study was to see how acupressure at distal acupuncture points of a specific meridian affects heart rate variability (HRV) and oxygenation of the trapezius muscle in healthy female subjects.

## 2. Methods

This study used a single-blind, randomized controlled trial. After receiving approval from the Nagoya Gakuin University Board of Ethics and obtaining written informed consent, 41 female participants were recruited for the present study. The subjects were randomly allocated to three groups. Exclusion criteria were conditions such as neck or shoulder pain, cardiovascular or neurological disease, diabetes, menstruation, or administration of sedatives, analgesics, or other medication.

**Figure 1:**
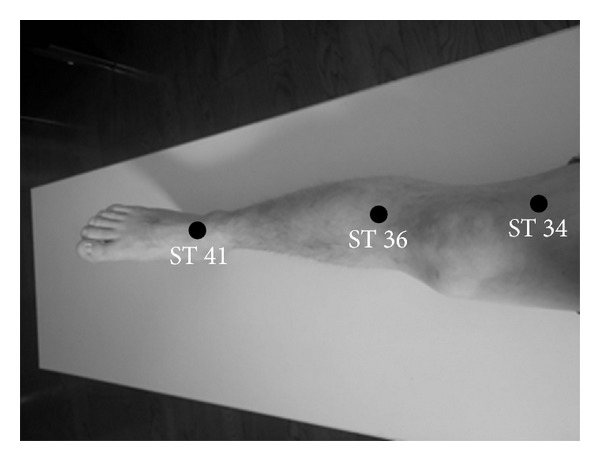
Three distal acupuncture points in the Stomach Meridian. ST 34, “Liangqiu,” ST 36, “Zusanli,” and ST 41, “Jiexi.”

**Figure 2:**
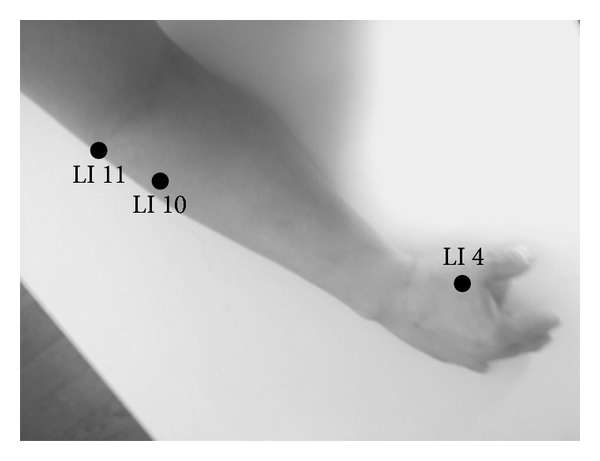
Three distal acupuncture points in the Large Intestinal Meridian. LI 4, “Hegu,” LI 10, “Shousanli,” and LI 11, “Quchi.”

Subjects in the Stomach Meridian acupuncture point (ST) group received acupressure at three distal acupuncture points, “Liangqiu” (ST 34), “Zusanli” (ST 36), and “Jiexi” (ST 41) (Figure 1), subjects in the Large Intestinal Meridian acupuncture point (LI) group received acupressure at three distal acupuncture points, “Hegu” (LI 4), “Shousanli” (LI 10), and “Quchi” (LI 11) (Figure 2), and subjects in the control group did not receive any stimuli.

All measurements were performed in the afternoon. Oxygenation of the trapezius muscles was measured using near-infrared spectroscopy (NIRS) (NIRO 200, Hamamatsu Photonics, Japan) bilaterally. Probes were placed at the transverse section on both sides of the upper trapezius muscles at the midpoint between the spinous process of the seventh cervical vertebra and the acromion. Measurements were given as concentration change in *μ*M of oxyhemoglobin (ΔO_2_Hb), deoxyhemoglobin (ΔHHb), and total hemoglobin (ΔTHb = ΔO_2_Hb + ΔHHb) from baseline [3, 9]. The electrocardiogram (ECG) signals were obtained from a portable ECG recorder (AC301A, GMS, Tokyo, Japan) and transferred to a computer loaded with HRV analysis software (TARAWA/WIN; Suwa Trust, Tokyo, Japan). The R-R intervals (RRIs) were obtained every 10 seconds. The two components of power of the RRI (ms·ms), low frequency (LF) (0.04–0.15 Hz) and high frequency (HF) (0.15–0.4 Hz), were calculated. The participants were allowed to sit comfortably on a chair in a quiet environment for 10 minutes. We then started recording oxygenation of the trapezius muscles and the ECG signal for HRV analysis. Three sets of acupressure were administered with the pulp of the right thumb in a rotary fashion at 20–25 cycles per minute for 30 seconds on each point on the right side of ST 34, ST 36, and ST 41 consecutively and afterwards on the left side of these three points in the ST group. Three sets of procedures conducted in the same way as the ST group were administered on the right side of LI 4, LI 10, and LI 11 consecutively and afterwards on the left side of these three points in the LI group. These procedures were performed by the same investigator. Following release of acupressure, the subjects were observed for another 10 minutes.

For data evaluation, the areas under the curve (AUC) of NIRS and HRV values were measured for the first rest period, each set of acupressure, and the second rest period [3, 10]. Data are presented as the median (range) or the median with the 25th and 75th percentiles. Data were analyzed using the Kruskal-Wallis test for intergroup comparison or the Friedman test for intragroup comparison followed by Dunn's method for multiple comparisons. A *P* value < 0.05 was considered statistically significant.

## 3. Results

Table 1 shows the demographic data of the three groups. There were no significant differences in age, height, and weight among the three groups.

**Figure 3:**
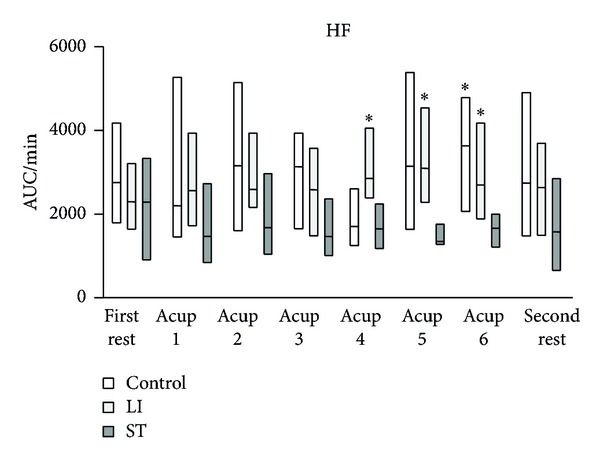
Change in the HF component of heart rate variability. AUC: area under the curve. Horizontal bars represent medians and boxes represent the 25th and 75th percentile ranges. * Different from the ST group (*P* < 0.05).

**Figure 4:**
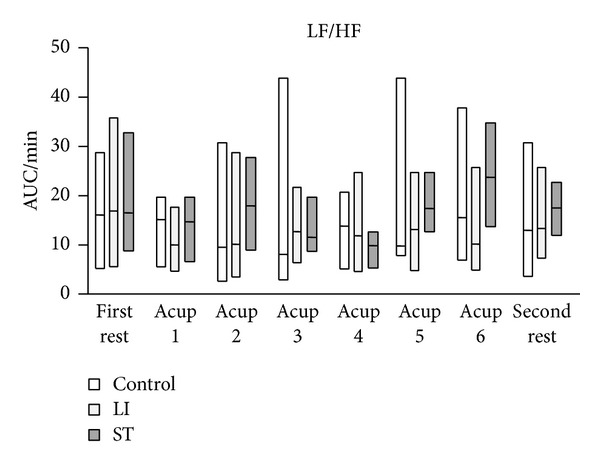
Change in the LF/HF ratio of heart rate variability. AUC: area under the curve. Horizontal bars represent medians and boxes represent the 25th and 75th percentile ranges.

**Figure 5:**
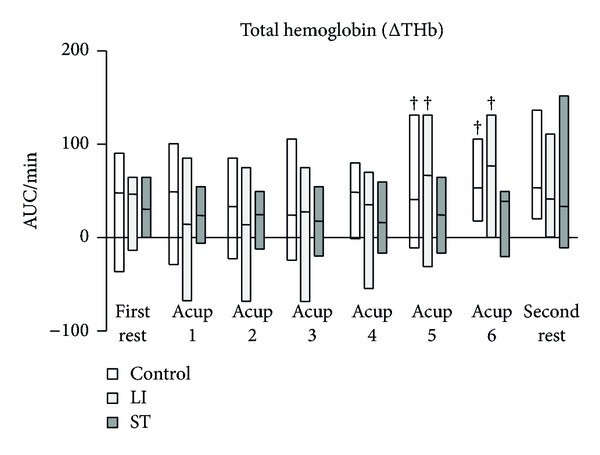
Change in total hemoglobin (ΔTHb) at the trapezius muscles. AUC: area under the curve. Horizontal bars represent medians and boxes represent the 25th and 75th percentile ranges. ^†^ Different from the first rest (*P* < 0.05).

**Figure 6:**
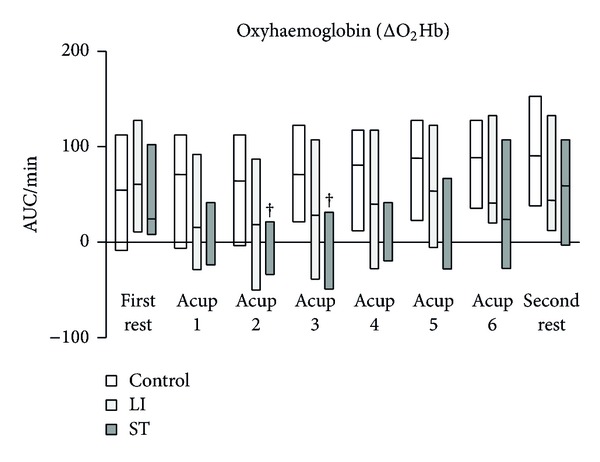
.Change in oxyhaemoglobin (ΔO_2_Hb) at the trapezius muscles. AUC: area under the curve. Horizontal bars represent medians and boxes represent the 25th and 75th percentile ranges. ^†^ Different from the first rest (*P* < 0.05).

The HF components of HRV in the control and LI groups tended to be higher than those in the ST group, and eventually the differences became significant (Figure 3). Although the LF/HF ratio of HRV had a tendency to increase in the ST group, there were no significant differences in the LF/HF ratio of HRV among the three groups (Figure 4). ΔTHb in the control and LI groups gradually increased and eventually reached significantly higher levels (Figure 5). In contrast, ΔTHb in the ST group did not change (Figure 5). While ΔO_2_Hb in the control and LI groups did not change, ΔO_2_Hb in the ST significantly decreased temporarily and returned to the baseline level during the acupressure manipulation (Figure 6).

## 4. Discussion

The present study showed that while the HF components of HRV in the control and LI groups tended to be higher than those in the ST group, the LF/HF ratio of HRV had a tendency to increase in the ST group. ΔTHb in the control and LI groups gradually increased and eventually reached significantly higher levels than those in the ST group. Also, ΔO_2_Hb in the ST group significantly decreased temporarily and returned to the baseline level during acupressure manipulation, while ΔO_2_Hb in the control and LI groups did not change.

Heart rate variability (HRV) has been used as a biomarker of autonomic nervous system function. HRV is a reliable method to obtain information on sympathetic and parasympathetic contributions to heart rate, and several studies have shown that pain increases sympathetic activity [11]. Frequency fluctuations in HRV in the range of 0.04–0.15 Hz (low frequency, LF) are considered to be markers of sympathetic and parasympathetic nerve activity, and high frequency (HF) fluctuations in the range of 0.15–0.4 Hz are considered to be markers of parasympathetic nerve activity. Thus, the LF/HF ratio is considered to be an index of sympathetic nerve activity [3, 11].

In the present study, the HF component of HRV in the ST group was lower than in the other groups and the LF/HF ratio of HRV had a tendency to increase in the ST group in contrast to the other groups, which means that sympathetic nervous activity increased in the ST group. We thus believe that acupressure at three distal acupuncture points in the Stomach Meridian, “Liangqiu” (ST 34), “Zusanli” (ST 36), and “Jiexi” (ST 41), led to sympathetic nervous activation. However, there is a possibility that stimulation caused by acupressure per se might have induced a sympathomimetic effect. In contrast, the HF component in the LI group was higher than that in the ST group and the LF/HF ratio in the LI group did not tend to increase, as observed in the control group, which means that parasympathetic nervous activity increased in the LI group. We thus believe that acupressure at three distal acupuncture points in the Large Intestinal Meridian, “Hegu” (LI 4), “Shousanli” (LI 10), and “Quchi” (LI 11), led to parasympathetic nervous activation in spite of acupressure stimuli.

ΔTHb in the control and LI groups eventually reached significantly higher levels than in the ST group. Furthermore, ΔO_2_Hb in the ST group significantly decreased temporarily during acupressure manipulation, while ΔO_2_Hb in the control and LI groups did not change. These data indicate that these three distal acupuncture points in the Stomach Meridian did not influence blood flow of the trapezius muscle but rather reduced oxygenation of the muscle. In contrast, these three distal acupuncture points in the Large Intestinal Meridian increased blood flow of the trapezius muscle and maintained oxygenation of the muscle.

Acupuncture is an important component of traditional Chinese medicine [12]. Over long periods of clinical practice, physicians have found that there is a specific effect of acupoints [13]; the specific effect may differ from acupoints on different meridians and meridians are, moreover, associated with specific body parts [12, 13]. These associations have been summarized and provide guidance for clinical practice. However, ancient laws should be verified by scientific methods. The present study showed that pressure stimulation on specific meridian or specific acupoint might differently affect HRV and blood flow and oxygenation of the trapezius muscle.

There is a limitation to the present study. The dermatomes of the thoracic region of the upper limb have their neuronal cell bodies in the same dorsal root ganglia and synapse on the same second order neurons in the spinal cord segments as the general visceral sensory fibers from the heart. The responses induced by acupuncture-like stimulation are reflexes in which cutaneous and/or muscle somatic afferent nerve fibers of the afferent limb, autonomic efferent nerve fibers of the efferent limb, and the spinal cord and/or the brain stem of the reflex centers work in a complex way [14]. Thus, stimulation of the arm region could theoretically affect heart rate. However, a previous study showed that acupuncture-like stimulation of forelimb, chest, abdomen, or hindlimb all decreased heart rate in rats [15]. We need to further evaluate the effect of nonacupoint stimulation on autonomic nervous activity and oxygenation of the trapezius muscle.

In conclusion, acupressure at three distal acupuncture points, LI 4, LI 10, and LI 11, maintained higher level of the HF components of HRV and blood flow and oxygenation of the trapezius muscle, compared with acupressure at three distal acupuncture points, ST 34, ST 36, and ST 41.

## Figures and Tables

**Table 1:** Demographic data.

	Control	ST	LI	*P*
Age (years)	20 (18–22)	20 (19–22)	21 (19–22)	0.108
Weight (kg)	50 (45–60)	55 (47–65)	50 (46–60)	0.108
Height (cm)	158 (150–168)	160 (157–172)	160 (150–166)	0.407

Values are median (range).
